# syN-BEATS for robust pollutant forecasting in data-limited context

**DOI:** 10.1007/s10661-024-13164-2

**Published:** 2024-10-02

**Authors:** Josef Berman, Ben Pinhasov, Moshe Tshuva, Yehudit Aperstein

**Affiliations:** Intelligent Systems, Afeka College of Engineering, Tel Aviv, Israel

**Keywords:** Pollution, Forecast, Meteorology, Deep learning, Ensemble models

## Abstract

This research introduces syN-BEATS, a novel ensemble deep learning model tailored for effective pollutant forecasting under conditions of limited data availability. Based on the N-BEATS architecture, syN-BEATS integrates various configurations with differing numbers of stacks and blocks, effectively combining weak and strong learning approaches. Our experiments show that syN-BEATS outperforms standard models, especially when using Bayesian optimization to fine-tune ensemble weights. The model consistently achieves low relative root mean square errors, proving its capacity for precise pollutant forecasting despite data constraints. A key aspect of this study is the use of data from only one meteorological and one air quality monitoring station per region, simulating environments with restricted monitoring capabilities. By applying this approach in regions with diverse climates and air quality levels, we thoroughly assess the model’s flexibility and resilience under different environmental conditions. The results highlight syN-BEATS’ ability to support the development of effective health alert systems that can detect specific airborne pollutants, even in areas with limited monitoring infrastructure. This advancement is crucial for enhancing environmental monitoring and public health management in under-resourced areas.

## Introduction

Industrial gross across both developed and developing nations has significantly escalated concerns regarding air quality and its consequential impact on public health and climate. High pollutant levels in densely populated areas are strongly associated with various health issues (Valavanidis et al., 2013; Smargiassi et al., 2009), highlighting the importance of effective air quality monitoring and forecasting systems.

Governments and environmental agencies around the world commonly employ the Air Quality Index (AQI) to measure and communicate the state of air quality. The AQI provides an aggregated assessment, consolidating various pollutants into a single indicative measure. This measure categorizes air quality levels from “good” to “hazardous,” based on the highest levels of specific pollutants normalized within predefined ranges (Kumari & Jain, 2018). However, a significant challenge in using AQI is the absence of a universally standardized definition. This lack of standardization results in variations in AQI calculation and interpretation across different countries (Ruggieri & Plaia, 2012; Monteiro et al., 2017), making it difficult to consistently compare air quality on a global scale.

Moreover, the AQI, due to its aggregate nature, lacks the granularity necessary to understand the specific risks associated with each pollutant. This is a critical gap, as different pollutants are linked to distinct health issues. For instance, high concentrations of sulfur dioxide ($SO_2$) have been associated with chronic asthma in children, while prolonged exposure to elevated ozone ($O_3$) levels is linked to pulmonary cancer (Valavanidis et al., 2013; Smargiassi et al., 2009). The ability to accurately identify and forecast concentrations of specific pollutants is therefore essential for effective public health management and targeted environmental policies.

In the field of pollutant forecasting, a myriad of research efforts have been undertaken, utilizing a wide array of methodologies. These range from traditional machine learning techniques (Donnelly et al., 2015; Corani & Scanagatta, 2016; Ghaemi et al., 2018; Ben Ishak et al., 2016; Prosdocimi et al., 2024) to sophisticated time series analysis methods (Chelani & Devotta, 2006; Díaz-Robles et al., 2008; Wang et al., 2017) and more recent advancements in deep learning models (Lin et al., 2018; Liao et al., 2020). However, a common limitation across many of these studies is their reliance on extensive spatial and temporal data, often sourced from a large network of air quality and meteorological monitoring stations. For example, Lin et al. (2018) utilized datasets for Los Angeles and Beijing, derived from 9 and 35 air quality monitoring stations, respectively. Similarly, Wang and Song (2018) employed data from 35 air quality stations along with 4 years of meteorological data, while Zhang et al. (2019) integrated data from 35 air quality monitoring stations with 18 meteorological stations over a 1-year period. Verma et al. (2024) perform spatio-temporal predictions of $NO_{2}$ concentrations in Delhi, India, using a bi-directional encoder with transformer on large amounts of spatio-temporal satellite data. The spatio-temporal distribution of atmospheric pollutants is governed by fluid dynamics principles. To accurately characterize the transport and dispersion of these pollutants, it is essential to capture the spatio-temporal meteorological properties across a given region. This necessitates the deployment of multiple meteorological and pollutant monitoring stations to effectively measure and analyze the convection patterns. It is important to note that the physico-chemical properties of the pollutants themselves can influence the convection processes, creating a complex interplay between pollutant characteristics and atmospheric dynamics. This dependency on large, multi-source datasets pose a significant challenge in regions where such extensive monitoring infrastructure is not available. The requirement for vast data collection from numerous stations limits the applicability of these models in many parts of the world where monitoring resources are scarce or non-existent. This scarcity of monitoring resources, particularly in less-developed regions, underscores a critical need in the field of environmental science: the development of adaptable and efficient forecasting models. These models must be capable of delivering reliable predictions even when working with datasets that are limited both in terms of size and diversity.

This challenge extends beyond a mere technical hurdle; it is about ensuring that all regions, irrespective of their infrastructure capabilities, have access to essential environmental health information. In response, the development of forecasting models for limited data scenarios has become a focal point in environmental research. To address the challenge of accurate forecasting with limited data, two main groups of methods and models have emerged. The first group involves using a single machine learning model to address the challenges of data scarcity, while the second is the use of ensemble models that leverage the collective strengths of multiple individual models to bolster forecasting reliability. In the first group of single models, Wang et al. (2021) demonstrated the efficacy of sparse functional multilayer perceptrons (SFMLP) for handling small samples in sparse time series data, showcasing its utility in scenarios where traditional dense sampling methods are inadequate. Sabo et al. (2023) demonstrated a deep learning model optimization for predicting crop yield in Algeria on limited data of vegetation index and local climate data, showing the benefit of shallow networks in cases of small data available for prediction. Chadoulos et al. (2023) introduced Deep4Ener, a single deep learning model designed for forecasting the energy demand of multiple consumers. This model leverages a novel neural network architecture, combining a GRU RNN encoder and an MLP, to accurately predict energy consumption across diverse consumer profiles, even in scenarios with limited data availability. A notable example of the ensemble-model approach is seen in the work of Abdulmajeed et al. (2020), where forecasts from diverse models such as ARIMA, Prophet, and Holt-Winters Exponential Smoothing were averaged to enhance prediction accuracy. Another example is averaging the weights of multiple identical neural networks with diverse weight initialization (Talaei-Khoei & Motiwalla, 2023).

In addition to single and ensemble methods, recent advancements have seen the emergence of hybrid models that integrate convolutional neural networks (CNNs), recurrent neural networks (RNNs), long short-term memory networks (LSTMs), and other statistical methods to enhance air quality index forecasting. For instance, Yan et al. (2021) utilized CNN, LSTM, and CNN-LSTM models alongside spatiotemporal clustering to enhance multi-hour and multi-site AQI forecasting in Beijing, showcasing methods that can adapt to the complexities of urban pollution dynamics. Similarly, Zhang and Li (2022) combined CNN and LSTM models to predict AQI in Beijing, demonstrating the potential of deep learning in handling the spatial-temporal aspects of air quality data. Another notable approach by Wang et al. (2022) involved a hybrid model that integrated CNN with an attention mechanism, highlighting the model’s capacity to focus on significant features amidst noisy environmental data streams. These studies, while not explicitly focused on sparse data environments, contribute valuable insights into handling data variability and gaps that are often present in real-world settings.

In the evolving landscape of time series forecasting, the N-BEATS model, introduced by Oreshkin et al. (2020), stands out with its deep learning architecture, designed for a broad range of applications. Its proven accuracy in various time series analyses marks a substantial leap in the field. Rajnish Rakholia et. al. demonstrated the use of N-BEATS with air pollutant concentration forecasting in Ho Chi Minh City, Vietnam, with accurate results relative to the stochastic nature of air pollutant dispersion (Rakholia et al., 2023). Our research seeks to explore how this SOTA model performs specifically in the realm of pollutant concentration forecasting under limited data conditions. We are particularly interested in determining whether N-BEATS can sustain its high performance with sparse datasets, a common challenge in environmental monitoring.

To address this, our study strategically utilizes data from just one meteorological station and one air quality monitoring station in a region. This decision is pivotal in demonstrating the feasibility of accurate pollutant forecasting in scenarios where data is scarce or monitoring infrastructure is limited. Our study focuses on two distinct regions, each characterized by unique climatic conditions, to evaluate the adaptability and effectiveness of our model across diverse environmental settings. This approach is supported by the use of high-quality, co-located meteorological and air quality data from each region, enhancing the reliability of our inputs. By testing the model in varied climates, we aim to comprehensively assess its performance and demonstrate its utility in scenarios where data availability is typically constrained. Central to our study is the development of the synergistic N-BEATS–syN-BEATS model, an innovative ensemble approach that leverages the strengths of various N-BEATS models. By combining models that function as both weak and strong learners, we aim to enhance the predictive performance beyond what single models can achieve. This architecture allows for a nuanced analysis that can accurately adapt to and predict pollutant levels under varied and limited data conditions. It represents a significant advancement in the field, showcasing that effective forecasting is achievable even with constrained data resources.

**Fig. 1:**
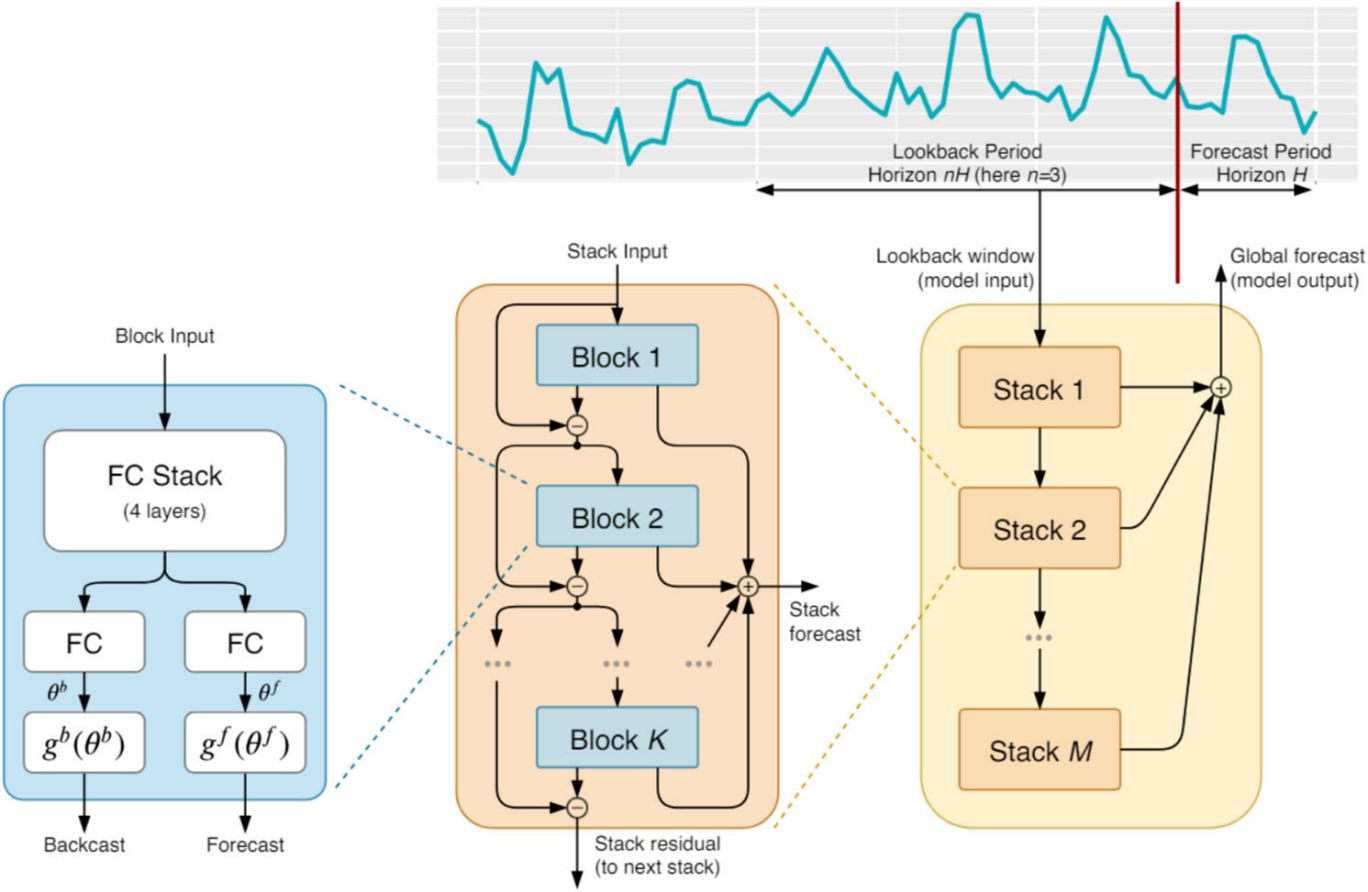
N-BEATS model architecture (Oreshkin et al., 2020)

## Contribution

This research advances the field of air quality forecasting by demonstrating the following key contributions:

- Introduction of the syN-BEATS model, an innovative ensemble adaptation of the N-BEATS architecture, specifically designed to enhance forecasting performance in scenarios with sparse datasets, a common hurdle in environmental monitoring.
- The study demonstrates a significant improvement in forecasting accuracy for air quality prediction with limited data using the syN-BEATS model compared to the baseline state-of-the-art N-BEATS model.
- We provide evidence of the syN-BEATS model’s adaptability and robustness across different climatic conditions. This underscores the model’s versatility in diverse environmental settings.
- Our findings show that an effective health alert system, capable of detecting individual airborne pollutants, can be developed in locations with only one meteorological and one air quality monitoring station. This is a significant advancement for public health and policy-making, especially in areas with limited monitoring resources.

## Methodology

### Deep learning models

Given the intricacies of pollutant concentration dynamics, coupled with significant challenges stemming from sparse and intermittent monitoring efforts in regions marked by diverse geographical landscapes, economic constraints, or infrastructural limitations, the adoption of advanced computational approaches becomes crucial. In this context, deep learning models, specifically designed to handle limited complex time series data, stand out as a promising solution to navigate through these multifaceted issues. This section will explore the N-BEATS model and its ensemble extension, the syN-BEATS model, offering a novel perspective to address the mentioned challenges.

#### N-BEATS model

N-BEATS has emerged as a notable model in the realm of time series forecasting, demonstrating the ability to capture and project significant time series behaviors effectively. Central to its design is its architectural composition of stacks and blocks.

Within this model, the primary unit of learning is the block. Each block learns from the time series data and calculates a forecast for a specific component of the time series. Successive blocks within a stack refine this forecast by working on the residuals—differences between the actual data and the forecast made by previous blocks. This approach ensures that each block learns the nuances missed by its predecessors.

The entirety of the model is structured in stacks, with each stack housing multiple blocks. Each stack sends out a forecast signal, which is a fraction of the model’s total output. All these stack signals are then aggregated to produce the final forecast. The visual representation of this model is depicted in Fig. 1.

For the purpose of our research, we specifically utilized the general architecture of the N-BEATS model. This version encompasses 30 stacks, each containing a single block. In this configuration, each stack acts as a weak learner, capturing broad patterns in the data without delving deep into detailed interpretations.

**Fig. 2:**
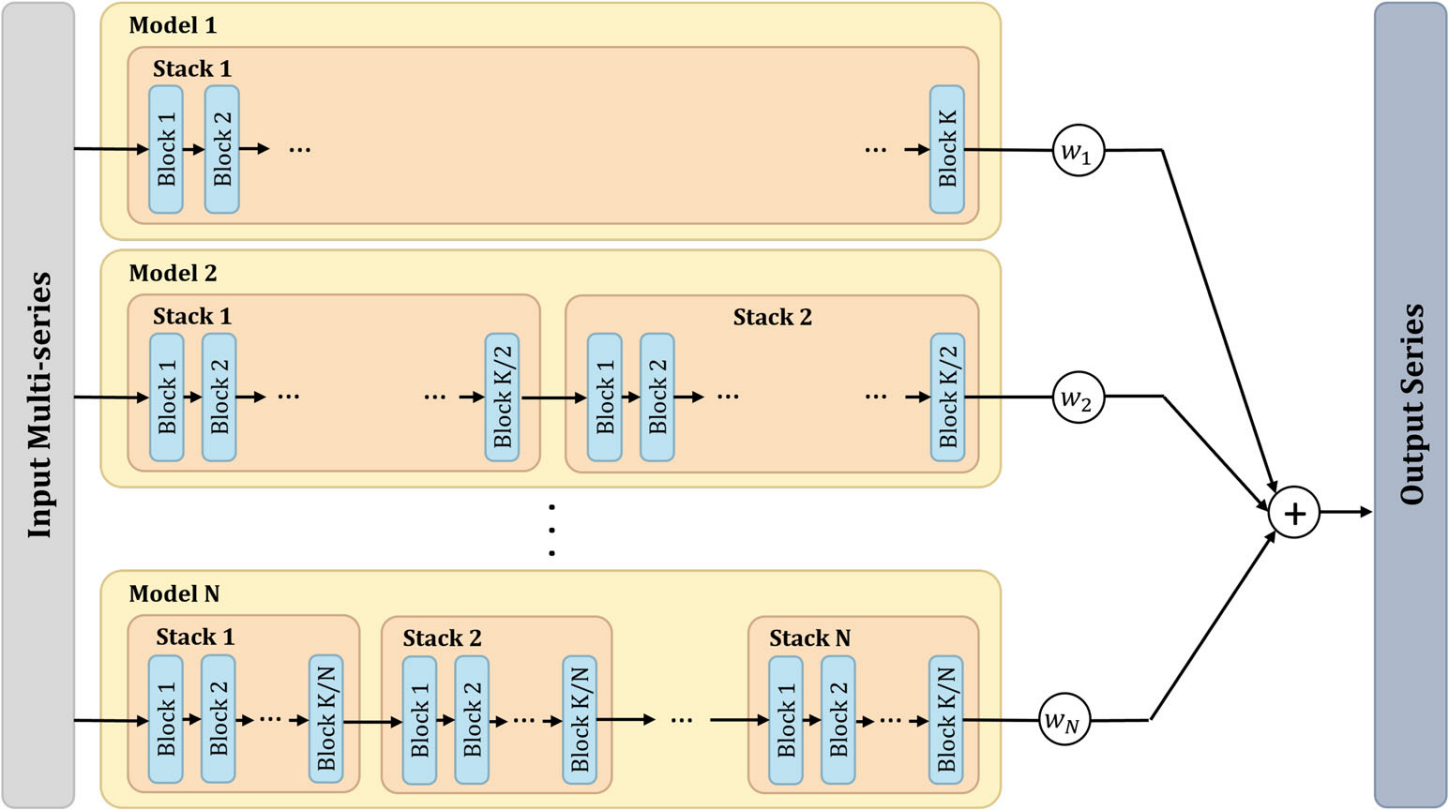
Architecture of syN-BEATS: ensemble of N-BEATS models with varying configurations of blocks and stacks, keeping a consistent total block count across configurations. The forecast of all configurations then linearly combined using a set of weights, either predefined or optimized

#### syN-BEATS model

In this paper, we introduce the synergistic N-BEATS–syN-BEATS model, an ensemble adaptation of the standard N-BEATS model, which brings together multiple N-BEATS configurations under one ensemble structure. This ensemble approach is designed to overcome the limitations of using a single model configuration, particularly in data-limited scenarios. While the standard N-BEATS model employs a single configuration of stacks and blocks, syN-BEATS combines multiple configurations with varying numbers of blocks and stacks. The architecture of the blocks within the stacks remains the same. This design enables syN-BEATS to balance the strengths of both weak and strong learners, providing a more robust and generalizable forecasting system. The motivation for developing syN-BEATS stems from the limitations observed in single-model approaches. Complex models with many blocks can closely fit training data, but they are prone to overfitting, especially with sparse datasets. Simpler models, with fewer blocks, may avoid overfitting but often fail to capture critical details. To address this, syN-BEATS incorporates weak learners, which excel at capturing broad stochastic patterns, alongside strong learners, which better fit training data but are less prone to overfitting. By leveraging both, syN-BEATS achieves greater accuracy and robustness, particularly when applied to pollutant forecasting in environments with limited data availability. The architecture of the syN-BEATS model is demonstrated in Fig. 2.

Models with numerous stacks, each with fewer blocks, serve as many weak learners. These configurations excel in handling stochastic data behaviors and are resistant to overfitting. However, they might not always align closely with ground truth data. Conversely, models with fewer stacks, each laden with more blocks, are analogous to strong learners. They can predict training data with high accuracy but are prone to overfitting, which might compromise their performance on unseen data. By amalgamating weak and strong learners, the syN-BEATS model harnesses the strengths of both. This ensemble approach seeks to offer forecasts with enhanced accuracy and robustness against overfitting. Once each model $j = 1,\ldots ,N$ in the ensemble produces its predicted value at time *t*, these forecasts $\hat{y}_{t,j}$ are aggregated linearly as described in Eq. 1,

1 $$\begin{aligned} y_t=\sum _{j=1}^{N}\hat{w}_j\cdot \hat{y}_{t,j} \end{aligned}$$

where the weights $\hat{w}_j$ are either predefined or optimized. In our experiments, we use syN-BEATS model consisting of N-BEATS models with all divisors of 30 as the number of stacks (1, 2, 3, 5, 6, 10, 15, and 30), where the corresponding number of blocks in each model is the complementary divisor with respect to 30. We investigate both scenarios: using predefined weights and weights that are optimized using Bayesian optimization.

### Evaluation metrics

In our study, which focuses on comparing the syN-BEATS model against the baseline N-BEATS model and SOTA ensemble models such as used by Abdulmajeed et al. (2020), we utilize two essential metrics for evaluation: Root Mean Square Error (RMSE) and Relative Root Mean Square Error (RRMSE). RMSE, a standard metric in predictive modeling, quantifies the average magnitude of prediction errors and serves as a foundational measure of model accuracy. Despite its utility, RMSE alone may not fully address the nuances of environmental data forecasting, particularly in the context of pollutants with varying concentration scales.

To objectively evaluate and compare the forecasting performance of the syNBEATS model against the N-BEATS model, we have selected the Relative Root Mean Square Error (RRMSE) as our primary evaluation metric. The choice of RRMSE is particularly salient in the realm of environmental studies, especially for pollutant predictions. It provides a measure of how close our predictions are to the actual observed values while also normalizing the error, making it relative and more interpretable across different scales of data.

The RRMSE is defined as

2 $$\begin{aligned} \text {RRMSE}=\frac{\sqrt{\frac{1}{n}\sum _{t=1}^{n}(y_t-\hat{y}_t)^2}}{\frac{1}{n}\sum _{t=1}^{n}y_t} \end{aligned}$$

where $y_t$ is the actual value at time *t*, $\hat{y}_t$ is the predicted value at time *t*, and *n* is the number of observations over time.

By adopting the RRMSE, we not only gauge the ensemble model’s forecasting accuracy but also assess its robustness and reliability. This is especially pertinent for ensemble models, where a balance between individual model contributions and their combined predictive power is critical.

## Dataset description

To develop a robust model for predicting concentrations of specific pollutants, our study strategically selects two regions with both climatic and AQI diversity. According to Köppen-Geiger climate classification, the first region, “Region A” is located in the “Csa” climate zone, indicating a Mediterranean climate with moderate temperatures and variable rainfall. The second region “Region B” falls under the “BWh” category, experiencing a hot desert climate marked by hot, arid conditions and significant temperature fluctuations. Also these regions differ in pollutant levels. Region A, being in close proximity to industrial areas, exhibits a higher AQI, indicative of greater pollutant concentrations resulting from industrial emissions.

Our methodology, by focusing on areas with both climatic and air quality diversity, is designed to thoroughly evaluate the model’s efficacy in diverse environments. To mirror limited data scenarios often encountered in environmental monitoring, we limit our data collection to one meteorological and one air quality monitoring station per region. This setup tests the model’s adaptability and robustness under scenarios of data scarcity.

Hourly pollutant concentration data used in this research is taken from the Israeli Ministry of Environmental Protection’s website (https://www.gov.il/en/departments/topics/reducing_air_pollution), over a time range of almost 3 years (March 9, 2020, to December 31, 2022), on five types of commonly monitored pollutants—nitrogen oxide (*NO*), nitrogen dioxide ($NO_2$), nitrogen oxide compounds (*NOx*), ozone ($O_3$), and small particulates ($PM_{2.5}$). Hourly meteorological data used in this research is taken from the Israeli Meteorological Service’s website (https://ims.gov.il/en/data_gov) over the same time period with 11 features (relative humidity; minimum, maximum, and average temperature; wind direction and speed; upper gust direction and speed; maximum minute wind speed; wind direction standard deviation; precipitation). Missing data in both datasets was interpolated using Akima interpolation method. Table 1 provides the dataset composition and statistical summaries for the common measures on meteorological data and pollution concentration.

**Table 1:** Common meteorology and pollutant concentration data statistics

		Temp	Relative	Wind	Precipitation	NO	$NO_2$	NOx	$O_3$	$PM_{2.5}$
		[$^\circ C$]	humidity	speed	[mm]	[$\mu g/m^3$]	[$\mu g/m^3$]	[$\mu g/m^3$]	[$\mu g/m^3$]	[$\mu g/m^3$]
			[%]	[*m*/*s*]						
Region A	count	24,593								
	mean	20.88	7.033	2.61	0.01	2.09	11.17	14.44	67.38	15.47
	std	6.99	18.73	1.56	0.14	2.99	9.72	12.11	29.62	11.02
	min	2.10	12.00	0.00	0.00	0.00	0.00	0.40	19.0	0.11
	25%	15.60	56.00	1.40	0.00	0.80	4.50	6.80	42.90	8.90
	50%	21.10	72.00	2.20	0.00	1.70	8.62	11.10	66.50	13.00
	75%	26.30	86.00	3.60	0.00	2.20	14.80	18.10	90.40	18.90
	max	42.70	100.00	14.70	7.60	123.10	120.70	262.80	180.50	355.60
Region B	count	24,612								
	mean	24.86	41.40	2.49	0.00	1.42	4.32	6.36	98.28	15.84
	std	8.24	18.38	1.20	0.03	1.10	2.56	3.75	19.85	15.64
	min	1.30	5.00	0.00	0.00	0.00	0.00	0.00	24.60	0.00
	25%	18.50	27.00	1.60	0.00	0.40	2.70	3.80	85.10	8.00
	50%	25.10	40.00	2.30	0.00	1.60	3.80	6.40	98.30	13.60
	75%	31.10	55.00	3.20	0.00	2.10	5.50	8.30	111.20	20.10
	max	47.20	95.00	16.60	2.50	52.40	35.10	91.40	191.60	416.40

## Experiments

This section details four experiments conducted to assess our forecasting models’ performance: the original N-BEATS model as a baseline, a baseline ensemble of statistical models, the syN-BEATS with predefined weights, and the syN-BEATS with optimized weights. All models were utilized using the Darts framework of forecasting models (Herzen et al., 2022). The neural models are trained on 120-h chunks of historic data to forecast 24 h ahead of each chunk.

We divided all collected data into training (76%), validation (19%), and testing (5%) sets, providing a uniform basis for evaluating the models’ predictive accuracy.

### Experiment 1: baseline single model

As originally constructed and optimized by Oreshkin et al. (2020) and adopted by Rakholia et al. (2023), the configuration of the baseline is using a single N-BEATS model composed of 30 stacks of one block each.

### Experiment 2: baseline ensemble model

Utilized by Abdulmajeed et al. (2020), a baseline ensemble model is used to compare the syN-BEATS’ performance with ensemble of statistical forecasting models. The baseline ensemble model consists of linear combination of forecasts of ARIMA (here utilized using AutoARIMA), Holt-Winters’ Exponential Smoothing, and Facebook Prophet.

### Experiment 3: syN-BEATS with predefined weights

syN-BEATS model was used to predict a 24-h forecast by using a linear combination of configuration-modified N-BEATS models, using weights predefined using the Relative Root Mean Squared Error of each model (where the training set of each model was further divided into training and validation sets in order to receive the validation error). The predefined weights are described in Eq. 3 as reference to the weights used in Eq. 2. Each weight is a normalized reciprocal of the squared RRMSE.

3 $$\begin{aligned} w_j=\frac{{1}/{\text {RRMSE}_j^2}}{\sum _{k=1}^N {1}/{\text {RRMSE}_k^2}} \end{aligned}$$

**Table 2:** Summary of RRMSE results comparing N-BEATS baseline model with the ensemble model–syN-BEATS

	Pollutant	Baseline	syN-BEATS model		syN-BEATS model	
		N-BEATS model	using predefined weights		using optimized weights	
		RRMSE	RRMSE	Relative error	RRMSE	Relative error
				reduction		reduction
Region A	*NO*	0.69	**0.31**	**55%**	**0.31**	**55%**
	$NO_2$	0.48	0.38	21%	**0.16**	**66%**
	*NOx*	0.44	0.32	27%	**0.15**	**65%**
	$O_3$	0.35	0.24	31%	**0.06**	**83%**
	$PM_{2.5}$	0.34	0.22	35%	**0.20**	**41%**
Region B	*NO*	0.48	0.19	60%	**0.17**	**65%**
	$NO_2$	0.42	0.37	12%	**0.29**	**31%**
	*NOx*	0.39	0.30	23%	**0.21**	**46%**
	$O_3$	0.12	0.07	42%	**0.04**	**67%**
	$PM_{2.5}$	0.66	0.44	33%	**0.21**	**68%**

### Experiment 4: syN-BEATS with optimized weights

Another method for determining the weights of the individual N-BEATS models is parameterization and tuning using a metric to determine the optimal weight values. The tuning was performed using Bayesian optimization with Mean Squared Error as the loss function. Bayesian optimization is an efficient method for tuning hyperparameters, where a surrogate model, typically a Gaussian process, is used to predict the objective function (Mean Squared Error in this case). It iteratively selects weight configurations based on a balance between exploring new possibilities and exploiting known good configurations. The optimization process adjusts the ensemble weights by evaluating different combinations and updating the surrogate model after each step. This method reduces the computational cost compared to traditional grid search, making it more suitable for complex models like syN-BEATS. The syN-BEATS model with optimized weights achieved lower RRMSE values relative to the other three models across both regions.

## Results

The results clearly demonstrate that the syN-BEATS model with optimized weights achieved lower RRMSE values relative to the other three models across both regions. This performance was particularly pronounced in Region A, which exhibits a “Csa” Mediterranean climate characterized by greater unpredictability in airborne pollutant concentrations. In Region A, the syN-BEATS model with optimized weights showed substantial reductions in RRMSE compared to the baseline N-BEATS model, as summarized in Table 2. Specifically, the RRMSE reductions ranged 41% for $PM_{2.5}$ to remarkable improvements of 68% for $PM_{2.5}$ and even 83% for $O_3$. The efficacy of the syN-BEATS model with optimized weights was also evident in Region B, which has a “BWh” hot desert climate typically associated with greater predictability. While the baseline statistical models and the N-BEATS model performed reasonably well in this region, the syN-BEATS model still demonstrated notable RRMSE reductions, ranging from 31% for $NO_2$ to 68% for $PM_{2.5}$, as shown in Table 2.

While the primary evaluation metric in this study was Relative Root Mean Square Error (RRMSE), to provide a clearer understanding of the error magnitude, we also evaluated the model performance using the Root Mean Square Error (RMSE) metric, which directly reflects the magnitude of prediction errors in the original pollutant concentration units. The RMSE values for the key pollutants are provided in Table 3. Relative error reduction is the same as in Table 2. RMSE results show a decrease in the order of magnitude of the concentration of the pollutants.

**Table 3:** Summary of RMSE results (${\mu g}/{m^3}$) comparing N-BEATS baseline model with the ensemble model–syN-BEATS

	Pollutant	Baseline	syN-BEATS model	syN-BEATS model
		N-BEATS model	using predefined weights	using optimized weights
Region A	*NO*	1.44	**0.65**	**0.65**
	$NO_2$	5.36	4.24	**1.79**
	*NOx*	6.35	4.62	**2.17**
	$O_3$	23.58	16.17	**4.04**
	$PM_{2.5}$	5.26	3.40	**3.09**
Region B	*NO*	0.68	0.27	**0.24**
	$NO_2$	1.81	1.60	**1.25**
	*NOx*	2.48	1.91	**1.34**
	$O_3$	11.79	6.88	**3.93**
	$PM_{2.5}$	10.45	6.97	**3.33**

**Fig. 3:**
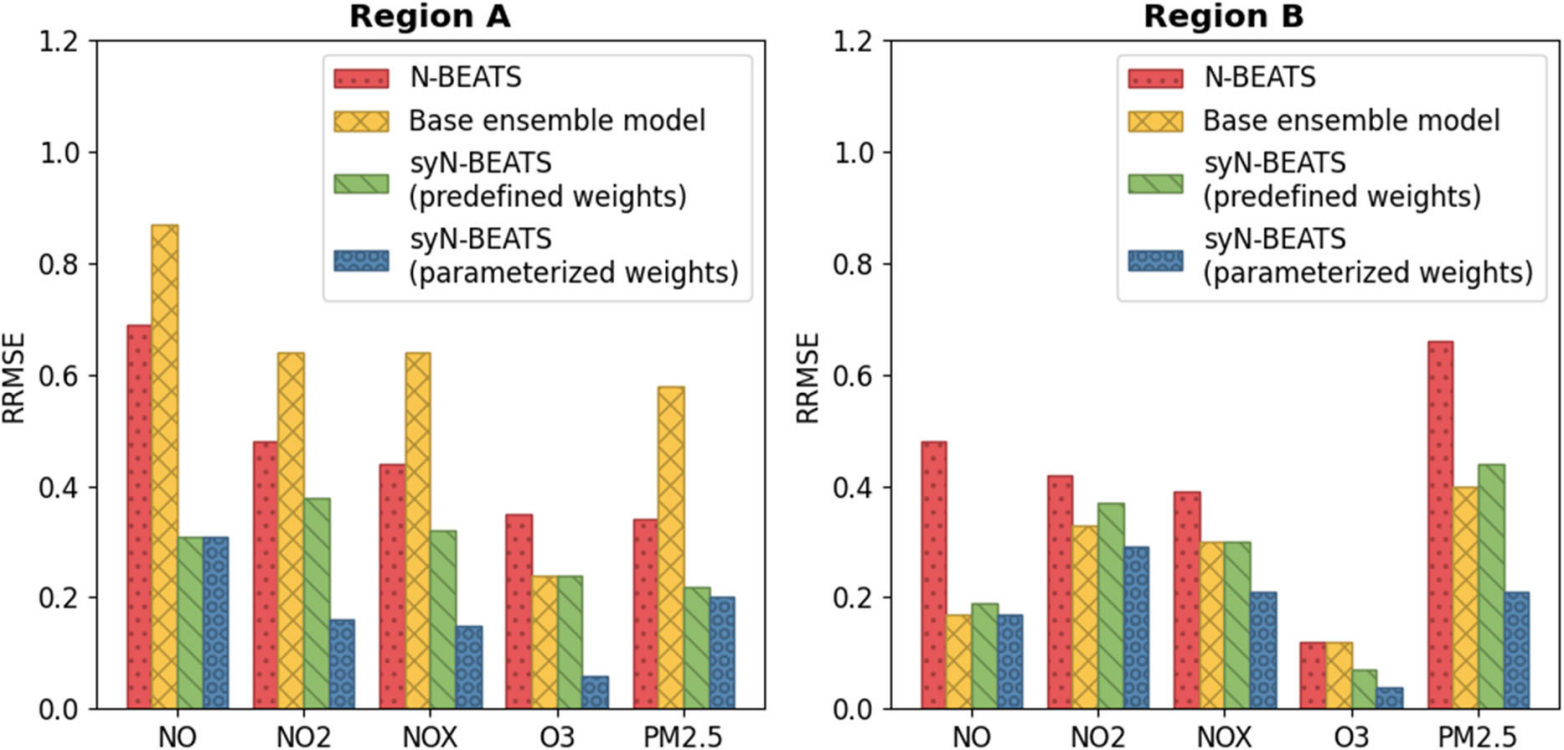
Comparison of the RRMSE of the four experiments

It is worth noting that the syN-BEATS model with predefined weights also outperformed the baseline N-BEATS model in both regions, albeit to a lesser extent compared to the optimized weights variant. This further underscores the advantages of the ensemble approach employed by syN-BEATS. The differences in performance between Region A and Region B can be attributed to the distinct climatic conditions of the two regions. Region A’s Mediterranean climate, with moderate temperatures and fluctuating rainfall, leads to greater variability in pollutant dispersion, making it a more challenging environment for accurate forecasting. The syN-BEATS model excelled in this region due to its ability to handle stochastic patterns and adapt to unpredictable pollutant behavior. In contrast, Region B’s hot desert climate, characterized by more stable and predictable pollutant levels, made the forecasting task somewhat easier for all models. However, even in this more predictable environment, syN-BEATS demonstrated significant improvements over the baseline, particularly in pollutants like $PM_{2.5}$ and $O_3$, indicating the robustness of the model across diverse environmental conditions (Fig. 3).

## Discussion

The results of this research demonstrate state-of-the-art forecasting capabilities for airborne pollutant concentrations using limited data from just one meteorological station and one air quality monitoring station per region. The proposed syN-BEATS model, an ensemble approach combining multiple configurations of the N-BEATS architecture, outperforms the baseline N-BEATS model by a significant margin when dealing with data scarcity scenarios. A key strength of the syN-BEATS model lies in its synergistic integration of weak and strong learners within the ensemble framework. By blending models with varying numbers of stacks and blocks, the ensemble can effectively capture both broad stochastic patterns and granular nuances present in the data. This combination enables syN-BEATS to deliver accurate forecasts while mitigating the risk of overfitting. The mitigation of overfitting is achieved by combining weak learners, which prevent overfitting by focusing on broader trends, with strong learners that capture finer details without overfitting to the training data. This balance ensures generalization across different datasets and avoids overfitting, even in complex environments like Region A. The performance gains achieved by syN-BEATS are particularly noteworthy in Region A, which exhibits a “Csa” Mediterranean climate characterized by greater unpredictability. In this region, the syN-BEATS model with optimized weights demonstrated up to an 83% reduction in Relative Root Mean Squared Error (RRMSE) compared to the baseline N-BEATS model. Even in Region B, with its more predictable “BWh” hot desert climate, syN-BEATS consistently outperformed the baseline models across various pollutants.

## Conclusion

These findings underscore the versatility and robustness of the syN-BEATS approach, showcasing its ability to adapt and deliver reliable forecasts across diverse environmental conditions—a crucial requirement for real-world applications in air quality monitoring and public health management. In addition, the Bayesian optimization used to tune the ensemble weights plays a significant role in mitigating overfitting. This automated process helps balance model complexity by optimizing the weight configurations, further ensuring that the model generalizes well without overfitting. Furthermore, the successful application of syN-BEATS using data from only one monitoring station per region demonstrates the model’s potential for developing effective health alert systems in areas with limited monitoring resources. This represents a significant advancement, as it enables the accurate detection and forecasting of individual airborne pollutants, even in regions where extensive monitoring infrastructure is lacking or nonexistent.

## Data Availability

Data is provided within the links implemented in the Dataset Description section in the manuscript, of the Israeli ministry of environmental protection, and the Israeli meteorological service.
